# Temporal dynamics of public transportation ridership in Seoul before, during, and after COVID-19 from urban resilience perspective

**DOI:** 10.1038/s41598-024-59323-w

**Published:** 2024-04-18

**Authors:** Sangwan Lee, Jooae Kim, Kuk Cho

**Affiliations:** LX Spatial Information Research Institute, Korea Land and Geospatial Informatix Corporation, 42, Jisaje 2-ro, Iseo-myeon, Wanju-gun, Jeollabuk-do Republic of Korea

**Keywords:** Public transportation, Transportation planning, COVID impact, Spatial difference-in-difference, Human mobility data, Psychology and behaviour, Socioeconomic scenarios

## Abstract

We delve into the temporal dynamics of public transportation (PT) ridership in Seoul, South Korea, navigating the periods before, during, and after the COVID-19 pandemic through a spatial difference-in-difference model (SDID). Rooted in urban resilience theory, the study employs micro-level public transportation card data spanning January 2019 to December 2023. Major findings indicate a substantial ridership decline during the severe COVID impact phase, followed by a period in the stable and post-COVID phases. Specifically, compared to the pre-COVID phase, PT ridership experienced a 32.1% decrease in Severe, followed by a reduced magnitude of 21.8% in Stable and 13.5% in post-COVID phase. Interestingly, the observed decrease implies a certain level of adaptability, preventing a complete collapse. Also, contrasting with findings in previous literature, our study reveals a less severe impact, with reductions ranging from 27.0 to 34.9%. Moreover, while the ridership in the post-COVID phase exhibits recovery, the ratio (Post/Pre) staying below 1.0 suggests that the system has not fully returned to its pre-pandemic state. This study contributes to the urban resilience discourse, illustrating how PT system adjusts to COVID, offering insights for transportation planning.

## Introduction

The COVID-19 pandemic has instigated profound transformations across various societal dimensions^1,2^, particularly impacting global public transportation (PT) systems^3^. As urban centers grapple with the unprecedented challenges posed by the virus, understanding the temporal heterogeneity of PT ridership becomes imperative. In this regards, a significant body of previous literature offers valuable insights into the immediate disruptions caused by the pandemic, revealing a dramatic decline in PT ridership and altered travel patterns^4–9^. These studies have shed light on the challenges faced by urban transportation during the acute phases of the pandemic. Nonetheless, there remains a critical void in our understanding of the comprehensive impact of COVID-19 across the entire spectrum of PT ridership—spanning from the period before the pandemic, through its duration, and into the post-COVID era^10,11^. While existing literature has provided valuable insights into the immediate disruptions caused by the pandemic, there remains a critical gap in comprehending the comprehensive impact of COVID-19 across the entire spectrum of PT ridership through its outbreak, duration, and into the post-COVID era. Amidst the transition to a post-COVID era, often referred as the “new normal,” there emerges a pressing need to explore evolving patterns and behaviors shaping the future of urban mobility.

This study attempts to establish a connection to urban resilience theory^12,13^, forming the conceptual framework that intricately engages with ongoing discussions in academic and policy spheres^14^. Adopting an urban resilience perspective offers a unique lens to examining how PT system can adapt, recover, and thrive in the face of disruptions like the COVID^15^. Specifically, urban resilience, characterized as the inherent capacity of transportation systems to either maintain or swiftly return to desired functions amid disturbances—a concept often encapsulated as “bounce back”—lays the foundation for theoretical advancements and practical applications. This is particularly crucial within the unique context of the COVID-19 pandemic. By integrating urban resilience theory into this research, we aim not only to uncover nuance temporal dynamics in PT ridership by also to gauge resilience, responsiveness, and adaptability within urban transportation systems. Positioned at this pivotal intersection, this study aims to bridge existing gaps in the literature by unveiling nuanced and evolving temporal patterns of PT ridership.

This study explores temporal variations related to COVID-19 in PT ridership using a big-data-driven temporal heterogeneity analysis, framed by Jia et al.^16^. Conducted in Seoul, a capital city renowned for its robust PT networks and ridership^17^, this research employs a rigorous methodological approach. We use personal card record data related to PT, including transit, subway, and bus services, collected over five years (January 2019 to December 2023) sourced from publically available repository of the Korea Transportation Safety Authority (TS). These micro-level data afford detailed insights into daily travel patterns^18^, enabling a comprehensive analysis of four distinct phases defined by National Institute of Infectious Disease in South Korea. These phases include the period before COVID outbreak (Pre), the phase during which severe COVID impact was experienced (Severe), the phase characterized by stable infection conditions during COVID (Stable), and the period following the official declaration of the end of the COVID era (Post). The data are derived from aggregated card usage, encompassing credit/debit cards and transit/bus passes, at monthly intervals. These records are also aggregated at the finest local administrative level, specifically Eup/Myeon/Dong (EMD), reflecting the aim of studying trends at the population level rather than focusing on individual behaviors. Moreover, employing spatial difference-in-difference models (SDID), the research delves into the temporal and distinct impact sizes of COVID-19 on PT ridership in Seoul, referred to as the treatment effect. The SDID model estimate the treatment effect while controlling for socio-economic and built environment characteristics, as well as seasonal factors known to significantly influence PT ridership, as demonstrated in previous literature^19–21^.

This research enhances our understanding of two key areas: firstly, it illuminates the evolving patterns of travel behavior before, during, and after the COVID-19 pandemic; secondly, it provides empirical insights into urban resilience through the examination of challenges faced by PT ridership during the COVID-19 crisis. By transcending the temporal constraints of the pandemic, this study offers a comprehensive view of PT ridership dynamics, thereby filling critical gaps in the existing literature pertaining to the post-COVID era. By underscoring the crucial role of transportation systems in urban resilience, this research enriches the ongoing discourse on how cities can not only withstand unforeseen disruptions but also flourish amidst change. Ultimately, this study contributes to a broader dialogue on urban resilience, emphasizing the significance of transportation systems in nurturing cities capable of enduring and thriving in dynamic and uncertain environments.

## Material and method

### Data sources

#### Human mobility data

This study utilizes publicly available human mobility data obtained from the Korea Transportation Safety Authority (TS), accessible at https://www.stcis.go.kr/wps/main.do. The data originate from individuals who voluntarily opt-in to share their location data anonymously through a framework compliant with the General Data Protection Regulation (GDPR), ensuring privacy standards are upheld. It is crucial to note that, in adherence to privacy concerns, only mobility-related data devoid of personal information or identifiers allowing user identification were employed in this research. Leveraging the micro-level mobility data, a relatively recent but invaluable resource in academia^22,23^, the research offers a detailed understanding of travel behaviors.

The datasets encompass monthly records of PT ridership, encompassing transit, bus, and subway, spanning from January 2019 to December 2023. These records are aggregated at the local authority level, specifically EMD, reflecting the aim of studying trends at the population level rather than focusing on individual behaviors. Seoul, as the study area, comprises a total of 422 EMDs, representing the finest administrative level in South Korea. The monthly records are meticulously collected and categorized by the hour, as well as origin/destination (OD), and subsequently aggregated at the EMD level.

To validate the representativeness of the data, an analysis was conducted by examining the correlation between the number of users and the population of EMDs across diverse age groups. This methodology aligns with established practices in previous literature, such as the work by Santana et al.^24^. The assessment revealed robust positive correlations across various age groups, including total, teenagers, working-age individuals, and the elderly. Notably, correlations of 0.730, 0.701, 0.734, and 0.697 were observed, all with a p-value less than 0.001, affirming the reliability and representativeness of the data for comprehensive analysis.

#### Other data sources

Additional data were gathered to serve as control variables in our model. Primarily, socio-economic data encompassing population statistics, housing prices, residential areas, and accessibility metrics to elementary schools, hospitals, and parks were obtained from the National Geographic Information Institute (NGII). These datasets, available for download at https://map.ngii.go.kr/mn/mainPage.do, provide essential contextual information for a comprehensive analysis. Moreover, to enrich our dataset, information on the locations of bus stops and subway stations was sourced from the Seoul Metropolitan Government datasets, accessible at https://data.seoul.go.kr/. Integrating these diverse datasets enhances the robustness of our analytical model, allowing for a more thorough exploration of the factors influencing PT dynamics in Seoul.

### Method

#### Spatial difference-in-difference

This study developed SDID to account for the spatial autocorrelation^25,26^ and enhance the accuracy of estimating the treatment effect^27,28^. Given the limitations of conventional a-spatial models, such as the ordinary least squares model (OLS), in controlling for spatial effects, potentially leading to biased and inconsistent outcomes^29^.

Our choice of SDID was motivated by the significant spatial dependency detected in residuals from OLS, as indicated by Moran’s I^30^ and Lagrange multiplier tests^31^. To select appropriate SDID model, we conducted a comparison between two spatial models, namely the spatial lag model (LAG) and spatial error model (ERR). The results of Akaike Information Criterion (AIC) and Log-Likelihood (LL) favored ERR as superior. Consequently, we determined ERR utilizing uniform kernel weight matrices to be the most suitable SDID for our dataset.

We conducted two validation test for our final model. First, we compared the results of OLS, LAG, and ERR to ensure the consistency of outcomes and validate our final model. Second, we conducted robustness tests using various types of spatial weight matrices. The outcomes of the two validation tests align with our finalized models, offering affirmation and validation for the effectiveness of our model.

#### Variables

The dependent variable in this study was the logarithmically transformed number of PT users each month over the 5 year period in Seoul. In further details, we explore variations in different times of the day, including both peak (i.e., 7–9 A.M. and 5–7 P.M.) and non-peak hours. These variations in ridership behavior have been previously documented in the literature^32^ and are integral to capturing the full spectrum of changes induced by external factors such as the COVID-19 pandemic. Moreover, we differentiate origin and destination of PT ridership. Thus, six dependent variables were utilized, encompassing the total number of PT users for origin (O_All), destination (D_All), origin during peak hours (O_Peak), destination during peak hours (D_Peak), origin during non-peak hours (O_Nonpeak), and destination during non-peak hours (D_Nonpeak). Consequently, six SDID models were developed, each corresponding to one of the dependent variables.

**Figure 1:**
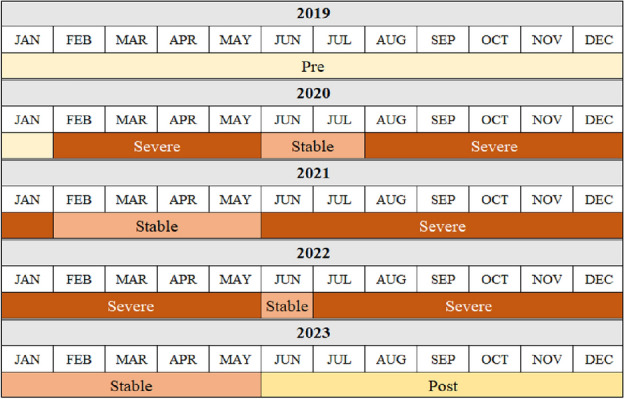
Classification of periods used in this study (Source: National Institute of Infectious Disease).

The primary focus of the models centered on estimating the parameters associated with a categorical variable that classified periods into four distinct phases (i.e., Pre, Severe, Stable, Post), referred to as the treatment effect. Specifically, this research classifies the COVID-19 timeline into the four specific stages, as depicted in Fig. 1. The classification was informed by reports from the National Institute of Infectious Disease, which classified periods into the four periods in this study based on the number of confirmed COVID cases and deaths.

To enhance precision, our models accounted for socio-economic, built environment, and seasonal effects known to significantly impact the number of PT users, as indicated by previous literature findings^19–21^. It is important to highlight that socio-economic and built environment characteristics may undergo significant changes over the five-year period. To address this temporal variation and ensure alignment with the corresponding time frames, distinct datasets were employed in our model.

#### Equation

The equation of our final SDID is as follows^33^:

$$log(y)={\beta }_{0}+{\beta }_{1}P+{\beta }_{i}{X}_{i}+\delta W\varepsilon +u$$

where $y$ denotes one of the dependent variables, ${\beta }_{1}$ indicates treatment effects, $P$ means a categorical variable of the four distinct phases of COVID, ${X}_{i}$ contains control variables used in this study, ${\beta }_{i}$ are their associated parameter estimates, and $u$ is the error term. $\delta$ denotes spatial error coefficient (lambda), $W$ indicates spatial weight matrix, and $\varepsilon$ is the error term.

## Results

**Figure 2:**
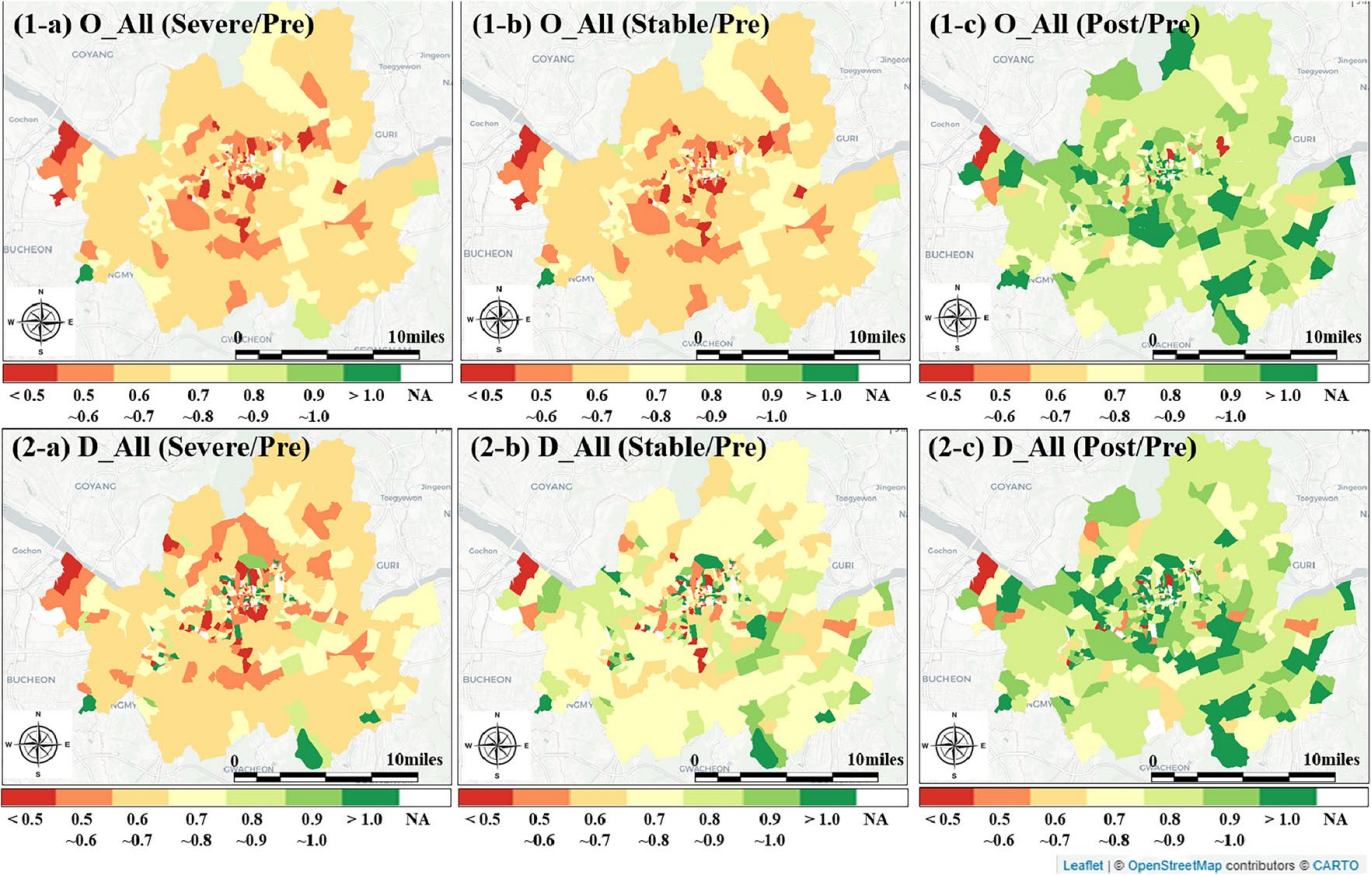
Temporal and spatial variations in public transportation ridership in Seoul using ratio (Note: The ratio is calculated by dividing the average number of ridership during Severe, Stable, and Post by that in the Pre period, which are defined as Severe/Pre, Stable/Pre, and Post/Pre. Also, the figures observe the temporal variations in two variables, which are O_All and D_All) (The maps in this figure were generated using a publically available package in R Studio 2023.03.0 + 386 called “Leaflet” and modified using Microsoft PowerPoint).

In this section, we delve into the temporal variations in PT ridership, encompassing transit, subway, and bus services. The first subsection compares the mean and median ridership across four distinct periods—Pre, Severe, Stable, and Post—in Table 1. Statistical significance of the mean differences is rigorously examined to provide deeper insights into the variations. The second subsection scrutinizes the changes in spatial dimensions, employing Fig. 2 to visually illustrate the shifting patterns. The third subsection offers findings of SDID models, particularly on the impact of COVID-19 on PT ridership.

**Table 1:** Temporal variations in public transportation ridership in seoul comparing mean and median across the four periods.

Variable	O_all	D_all	O_peak	D_peak	O_nonpeak	D_nonpeak
Descriptive statistics						
Period	Mean (median)	Mean (median)	Mean (median)	Mean (median)	Mean (median)	Mean (median)
Pre	802,640 (299,842)	789,765 (284,798)	254,123 (91,555)	235,660 (82,942)	528,463 (199,698)	536,969 (194,632)
Severe	611,322 (228,782)	604,845 (221,667)	205,731 (72,292)	195,505 (69,367)	388,767 (149,518)	394,693 (145,462)
Stable	646,319 (243,082)	639,690 (234,945)	216,256 (76,410)	206,199 (72,608)	412,228 (158,872)	418,119 (154,350)
Post	713,811 (276,471)	706,295 (264,840)	232,741 (82,777)	223,400 (79,247)	461,713 (181,536)	466,032 (179,009)
Bivariate statistics						
P-value of ANOVA	< 0.001	< 0.001	< 0.001	< 0.001	< 0.001	< 0.001

Variables: The total number of public transportation users for origin (O_All), destination (D_All), origin during peak hours (O_Peak), destination during peak hours (D_Peak), origin during non-peak hours (O_Nonpeak), and destination during non-peak hours (D_Nonpeak). Periods: the period before COVID outbreak (Pre), the phase during which severe COVID impact was experienced (Severe), the phase characterized by stable infection conditions during COVID (Stable), and the period following the official declaration of the end of the COVID era (Post).

### Temporal trends in public transportation ridership

Table 1 provides an overview of the descriptive and bivariate analysis results, elucidating the temporal patterns observed in each of four periods for six key variables related to PT ridership. The analysis reveals distinct temporal variations across the periods. Specifically, before the COVID outbreak (Pre), the mean number of PT ridership for origin (O_All) was 802,640, with a median of 299,842, indicating a high level of PT ridership. During the severe COVID impact phase, this mean decreased significantly to 611,322, with a median of 228,782, signifying a sharp decline in PT ridership. In the stable phase, the mean slightly increased to 646,319, with a median of 243,082, indicating relatively stable PT ridership. Finally, during the post-COVID period, the mean further increased to 713,811, with a median of 276,471, pointing to a recovery in PT ridership.

The pattern observed in the O_All column illustrates a clear trend of decreasing PT ridership during the severe COVID impact phase, followed by a recovery during the stable and post-COVID periods. ANOVA results further confirm significant variations in ridership across different periods, underscoring the impact of the COVID outbreak on PT ridership in Seoul, South Korea. The substantial decrease in PT ridership during the severe COVID impact phase highlights the immediate and severe impact of the pandemic on commuter behavior and mobility patterns in Seoul. Also, the observed recovery in PT ridership during the stable and post-COVID periods suggests a resilient capacity of the transportation system to adapt and regain users. Additional finding from the table is the consistency in origin/destination and peak/non-peak hour patterns despite the overall fluctuations in PT ridership over the different phases, suggesting a certain level of stability in travel behavior across these specific dimensions.

### Spatial and temporal trends in public transportation ridership

Figure 2 illustrates the spatial and temporal trends in PT ridership throughout the four periods using ratios, calculated by dividing the average number of ridership during Severe, Stable, and Post by that in the Pre (Severe/Pre, Stable/Pre, and Post/Pre). There are several major findings. First, the results reveal distinct spatial patterns in Severe/Pre, Stable/Pre, and Post/Pre, with increasing ratios indicating a continuous rise in PT ridership in Severe, Stable, and Post compared to the Pre period. Second, the results reveal a significant decrease in PT ridership during Severe compared to Pre, particularly in Seoul’s inner-city areas connected to the regional rapid transit network in South Korea, suggesting that these regions were more severely impacted during the height of the pandemic. Third, the recovery in PT ridership during the Stable period, especially in EMDs containing major park infrastructures, suggests that these areas experienced a quicker rebound. This insight could be leveraged to identify factors contributing to resilience, potentially guiding strategies to enhance transportation resilience in other regions. Fourth, Fig. 2 (2-b) indicates a more substantial recovery in PT ridership across EMDs in Seoul during the Stable period compared to Severe. Notably, more than 5 EMDs even show increased ridership compared to the Pre period. Finally, the majority of EMDs exhibit a ratio of more than 0.8 between Post and Pre, signifying the rebound in PT ridership after COVID. However, approximately 10 out of 422 EMDs demonstrate a ratio of more than 1.0, indicating increased ridership, while the majority experience a decrease.

### Covid effects on public transportation ridership

Table 2 presents the results of SDID models that quantify PT ridership changes in four distinct phases (i.e., Pre, Severe, Stable, Post). The primary focus is on the parameter estimates of a categorical variable representing the treatment effect, categorizing the periods into these four phases. Table 2 shows that SDID 1 and SDID 2 models are align with the findings in Table 1, revealing that, compared to the Pre phase, PT ridership experienced a 32.1% decrease in Severe, followed by a reduced magnitude of 21.8% in Stable and 13.5% in Post. Similar effect sizes are observed in SDID 2, with reductions of 31.6% in Severe, 20.8% in Stable, and 13.3% in Post. However, during peak hours (07:00– 09:00 and 17:00– 19:00), the effect sizes are comparatively lower, as indicated in SDID models 3 and 4. Conversely, during non-peak hours, the magnitudes of ridership decrease during and post-COVID eras align with those in the first and second SDID models.

**Table 2:** Quantifying COVID effect sizes on public transportation ridership in Seoul using spatial difference-in-difference models.

Model	SDID 1	SDID 2	SDID 3	SDID 4	SDID 5	SDID 6
Variable	O_All	D_All	O_Peak	D_Peak	O_NonPeak	D_NonPeak
Constant	15.939***	16.962***	19.064***	19.516***	13.042***	14.528***
Lambda	0.982***	0.983***	0.981***	0.983***	0.984***	0.983***
COVID period (reference. pre)						
Severe	− 0.321***	− 0.316***	− 0.271***	−0.270***	− 0.349***	− 0.342***
Stable	− 0.218***	− 0.208***	− 0.172***	− 0.168***	− 0.243***	− 0.230***
Post	− 0.135***	− 0.133***	− 0.120***	− 0.122***	− 0.143***	− 0.141***
Control variables						
Population	− 0.444***	− 0.339***	− 0.335***	− 0.211***	− 0.507***	− 0.420***
Bus	0.015***	0.015***	0.015***	0.018***	0.016***	0.013***
Subway	0.014	0.022**	0.024**	0.031***	0.007	0.017
Price	− 0.540***	− 0.525***	− 0.683***	− 0.669***	− 0.461***	− 0.451***
Residential	0.955***	0.922***	0.856***	1.011***	1.032***	0.875***
Area	1.578***	1.644***	1.704***	1.963***	1.523***	1.469***
Elementary	2.602***	2.555***	2.921***	2.964***	2.442***	2.313***
Hospital	− 0.480***	− 0.554***	− 0.497***	− 0.634***	− 0.481***	− 0.523***
Park	− 1.888***	− 1.869***	− 2.212***	− 2.262***	− 1.719***	− 1.653***
Seasonal factors						
Year (reference. 2019)						
2020	0.006	0.01	−0.003	0.019	0.011	0.004
2021	0.157*	0.149**	0.140*	0.160**	0.167**	0.144*
2022	0.274***	0.262***	0.241***	0.262***	0.292***	0.265***
2023	0.323***	0.314***	0.278***	0.300***	0.346***	0.324***
Month (reference. JAN)						
FEB	0.019	0.032	0.016	0.042	0.02	0.027
MAR	− 0.174***	− 0.167***	− 0.173***	− 0.176***	− 0.169***	− 0.160***
APR	0.066**	0.063**	0.062**	0.065**	0.067**	0.062**
MAY	0.113***	0.105***	0.114***	0.106***	0.111***	0.104***
JUN	0.088***	0.075***	0.090***	0.075***	0.088***	0.075***
JUL	0.156***	0.143***	0.155***	0.147***	0.155***	0.141***
AUG	0.117***	0.106***	0.082***	0.073***	0.137***	0.124***
SEP	0.137***	0.132***	0.142***	0.138***	0.134***	0.128***
OCT	0.061**	0.057**	0.057*	0.050*	0.063**	0.061**
NOV	0.031	0.03	0.017	0.014	0.039	0.038
DEC	− 0.013	− 0.013	− 0.011	− 0.012	− 0.014	− 0.015
Model statistics						
N	21,840	21,840	21,840	21,840	21,840	21,840
LL	− 1,315.03	− 1,142.17	− 1,326.40	− 1,193.25	− 1,316.78	− 1,148.59
AIC	2,690.05	2,344.34	2,712.81	2,446.51	2,693.55	2,357.17

Dependent Variables: log-transformed total number of public transportation users for origin (O_All), destination (D_All), origin during peak hours (O_Peak), destination during peak hours (D_Peak), origin during non-peak hours (O_Nonpeak), and destination during non-peak hours (D_Nonpeak). Control Variables: log-transformed population density (Population), the number of bus stops (Bus), the number of subway stations (Subway), log-transformed average housing sale price (Price), log-transformed total area of using residential purpose (Residential), log-transformed area of EMDs (Area), log-transformed network distance to the nearest elementary school (Elementary), log-transformed network distance to the nearest hospital (Hospital), and log-transformed network distance to the nearest park (Park). Periods: the period before COVID outbreak (Pre), the phase during which severe COVID impact was experienced (Severe), the phase characterized by stable infection conditions during COVID (Stable), and the period following the official declaration of the end of the COVID era (Post). Spatial difference-in-difference model (SDID), dependent variable (DV), the number of observations (N), log-likelihood (LL), and Akaike Information Criterion (AIC). The spatial dimension for all variables consists of EMDs.

Turning to the control variables, notable findings emerge. Unlike previous literature^34^, higher population density at the EMD level corresponds to lower PT ridership during both peak and non-peak hours. In the context of the COVID-19 pandemic, areas with higher population density might have experienced greater concerns about social distancing and public health. Residents in densely populated areas may have been more inclined to avoid crowded PT, contributing to the observed decrease in PT ridership. The impact is likely amplified during peak hours when congestion and close contact are more prevalent. Additionally, improved transportation accessibility to elementary schools exerts a negative influence on PT ridership. Enhanced accessibility to elementary schools may indicate better local infrastructure and amenities, making residents less reliant on PT for daily commuting. Other factors, consistent with previous literature^19,20,35^, indicate that higher housing prices in EMDs are associated with lower PT ridership (parameter estimates of − 0.540, − 0.525, − 0.683, − 0.669, − 0.461, and − 0.451). Areas with higher housing prices may cater to a demographic with a higher income level that prefers private transportation or resides closer to workplaces and amenities, which is a similar to a long-standing knowledge^36^.

## Discussions

**Figure 3:**
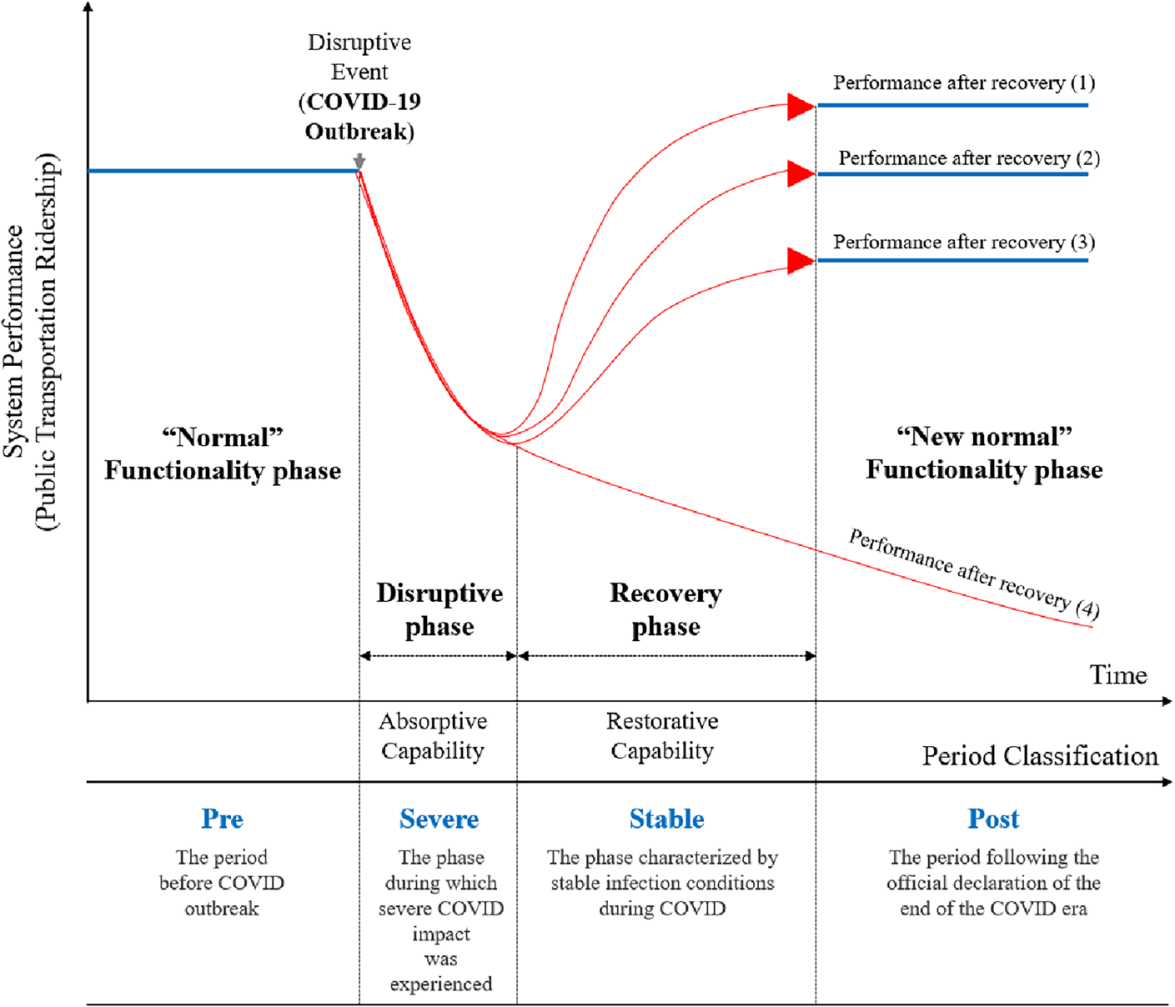
Urban resilience graph (Note: this graph is reformulated based on the previous literature of Koren et al.^37^).

A notable addition of urban resilience theory in this study is the adoption of a resilience function graph, depicted in Fig. 3, which visually captures how cities respond to disruptions. This graph serves as a dynamic tool, showcasing a spectrum from collapse to adaptation and the ideal “bounding back”—a reduction in disruption magnitude. Thus, this research leverages the resilience graph as an analytical tool, providing a tangible representation of how cities navigate disruptions, adapt, and ideally “bound back” over time.

**Figure 4:**
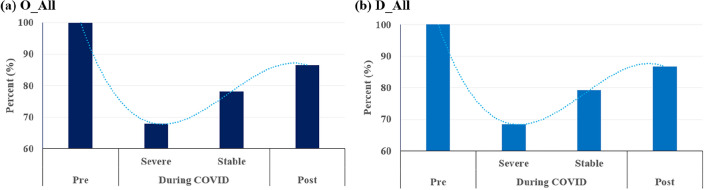
Application of urban resilience graph to temporal patterns in public transportation ridership estimated by spatial difference-in-difference models (Note: the illustrations depict public transportation ridership as 100% during the pre-COVID phase, while the figures during the severe, stable, and post-COVID periods are based on treatment effects computed through the spatial difference-in-difference model. These values are then integrated into the urban resilience graph for visual representation.).

The resilience graph, as applied to the major findings of this study, provides valuable insights into the adaptive capacity of Seoul’s PT system across distinct phases of the COVID-19 pandemic—Pre, Severe, Stable, and Post (see Fig. 4). First, approximately 32% decline in ridership during the Severe phase is indicative of the system facing a severe disruption, momentarily straining its resilience. Interestingly, the observed decrease implies a certain level of adaptability, preventing a complete collapse. This adaptability could be attributed to several factors. Seoul, known for its efficient and intricate public transportation network, might have implemented responsive strategies to maintain service continuity despite the disruptions caused by the pandemic. Moreover, the adaptability might reflect the city’s effective communication of safety measures, encouraging residents to continue using public transportation for essential travel while adhering to guidelines.

Regarding COVID effect size comparison with previous studies, the impact of the COVID-19 outbreak on PT ridership has been substantial, resulting in significant ridership reductions of 60 to 95% in major cities worldwide, such as New York, Singapore, and Toronto^38,39^. However, contrasting with these global trends, our study in Seoul reveals a less severe impact, with reductions ranging from 27.0 to 34.9%. This discrepancy underscores the variability in the COVID influence on PT across diverse cities. More importantly, our findings diverge from previous literature by finding a certain level of adaptability within PT system in Seoul. Rather than experiencing a complete collapse, our results suggest a partial recovery from the disruptive event, suggesting the ability of PT to adapt to challenging circumstances from the perspective of urban resilience.

Second, the relatively stable ridership (around 20% reduction in PT ridership) during Stable period signifies the system’s ability to adapt and find a new equilibrium. It demonstrates a rebounding effect, indicating a partial “bounding back” from the disruptions experienced during the severe impact phase.

Lastly, while the ridership in the post-COVID phase exhibits recovery, the ratio staying below 1.0 suggests that the system has not fully returned to its pre-pandemic state (approximately 13% reduction in SDID models). The graph illustrates ongoing challenges and highlights the need for continued adaptability. In essence, this research asserts that the temporal trajectory of PT ridership aligns with the performance depicted by the “Performance after Recovery (3)” route in Fig. 3, signifying a scenario of partial recovery from COVID.

The findings of this study carry several policy implications that can inform strategic interventions and decision-making in the realm of urban transportation. The temporal analysis of PT ridership during distinct phases of the COVID-19 pandemic provides a basis for targeted policy considerations. First, the significant decline in PT ridership during the severe COVID impact phase highlights the need for transportation planners to develop more resilient and adaptable transportation systems that can withstand external shocks such as pandemics. Second, in response to the COVID impact on travel patterns, transportation planners could explore innovative solutions such as dynamic routing and scheduling algorithms that can adapt to changing demand patterns and provide efficient and responsive transportation services throughout the day. Third, the slight decrease in PT ridership during the post-COVID era may be linked to the widespread adoption of remote work and altered commuting patterns. Policymakers and urban planners should consider these shifts when designing transportation strategies to align with the evolving needs and preferences of the population. Fourth, the slight decrease in ridership indicates that PT infrastructure needs to adapt to the changing demands of the post-pandemic population. This may involve reevaluating routes, frequencies, and modes of transportation to better serve the needs of commuters in the altered urban landscape. Fifth, encouraging alternative transportation modes, such as cycling, walking, or shared mobility options, could be considered to supplement PT and address the decreased ridership. Integrated and sustainable transportation solutions should be explored to offer diverse options to the public.

In addition to the implications on major findings, we offer additional discussion on human mobility data, derived from our routines such as frequent locations, purchases, and social interactions, creates a digital record of daily activities^24,40^. Decoding and analyzing this digital trail offer a novel platform for understanding urban dynamics, particularly when using card data from PT^41^. The sequence of locations captured through PT card use provides a unique opportunity to assess people’s urban activities in almost real time, overcoming limitations of traditional surveys and censuses^24^. By utilizing PT card data, this research transforms it into continuous snapshots of individual mobility patterns, serving as a crucial tool for gaining insights into population dynamics during situations that demand rapid responses. The use of such detailed data not only enhances the precision of the analysis but also contributes to the robustness of findings, providing a valuable resource for policymakers, urban planners, and transportation management. Moreover, the micro-level perspective enables the identification of subtle variations in ridership patterns, offering a more comprehensive and context-specific understanding of the necessity of incorporating this kind of data in transportation planning, particularly in the dynamic context shaped by the COVID-19 pandemic in Seoul, South Korea.

## Concluding remarks

Understanding the temporal dynamics of PT ridership is paramount for urban planners and policymakers seeking to create adaptive, resilient, and responsive transportation systems. By delving into the temporal heterogeneity of PT ridership in Seoul from urban resilience perspective, this study goes beyond a surface-level examination of pandemic-induced disruptions, offering an in-depth analysis of how PT system in Seoul coped with the crisis. This resilience assessment serves as a foundation for identifying strengths, vulnerabilities, and areas for improvement, guiding efforts to build resilient urban transportation systems.

While this research contributes valuable insights into the temporal dynamics of PT ridership in the context of the COVID-19 pandemic, it is essential to acknowledge certain limitations. First, the study relies on publicly available human mobility data, specifically from individuals who have voluntarily opted to share their location data anonymously. This voluntary opt-in nature may introduce a selection bias, as those who choose to participate may differ in certain characteristics from those who do not, impacting the generalizability of the findings. Second, the study is confined to the geographical scope of Seoul, South Korea. While Seoul serves as a rich and diverse case study, the findings may not be directly applicable to other urban contexts with distinct socio-economic, cultural, and infrastructural characteristics. Generalizing the results beyond Seoul should be done cautiously, considering the unique attributes of different metropolitan areas. Third, the research focuses solely on PT card data, which, while offering a detailed perspective on travel behaviors, may not capture the entirety of individuals’ mobility patterns. Fourth, temporal scope of this study is bounded by the available data from January 2019 to December 2023. While this period encompasses the pre-COVID, severe COVID impact, stable, and post-COVID phases, it does not extend beyond the provided timeframe. Future research with longer temporal coverage could provide a more extensive view of the lasting impacts of the pandemic on PT dynamics. Fifth, due to the data limitation, this study does not differentiate PT into specific modes, such as subway, bus, and transit. Lastly, the mobility data utilized in our study was collected anonymously from card records, limiting our ability to explore how demographic characteristics, social behaviors, and economic conditions intersect with the temporal dynamics of PT ridership.

## Data Availability

The datasets used and/or analyzed during the current study available from the corresponding author on reasonable request. Also, it is noted that all data used in this research are deposited in public repository. Data on public transportation cards were acquired from the Korea Transportation Safety Authority (TS), which may be found at https://www.stcis.go.kr/wps/main.do (accessed on January 10, 2024). The National Geographic Information Institute (NGII) provided the socio-economic data. The files can be downloaded at the website https://map.ngii.go.kr/mn/mainPage.do (accessed on November 20, 2023). The data regarding the locations of bus stops and subway stations was obtained from the datasets provided by the Seoul Metropolitan Government, which may be accessed at https://data.seoul.go.kr/. (accessed on December 1, 2023).
